# The Cross-Sectional Association between Consumption of the Recommended Five Food Group “Grain (Cereal)”, Dietary Fibre and Anthropometric Measures among Australian Adults

**DOI:** 10.3390/nu9020157

**Published:** 2017-02-18

**Authors:** Flavia Fayet-Moore, Peter Petocz, Andrew McConnell, Kate Tuck, Marie Mansour

**Affiliations:** 1 Nutrition Research Australia, Level 13, 167 Macquarie St, Sydney NSW 2000, Australia; andrew@nraus.com (A.M.); kate@nraus.com (K.T.); mariegmansour@gmail.com (M.M.); 2 Department of Statistics, Macquarie University, Sydney NSW 2000, Australia; peter.petocz@mq.edu.au

**Keywords:** grain, cereal, fibre, BMI, waist circumference, dietary guidelines

## Abstract

The Australian Dietary Guidelines recommended “grain (cereal)” core food group includes both refined and whole grain foods, but excludes those that are discretionary (i.e., cakes). We investigated the association between daily serves from the “grain (cereal)” group and its effect on fibre and adiposity. Data from Australian adults in the 2011–2012 National Nutrition and Physical Activity Survey were used (*n* = 9341). Participants were categorised by serves of core grain foods and general linear models were used to investigate the effect of demographic, socioeconomic, and dietary covariates on waist circumference, body mass index (BMI) and fibre intake. Compared to core grain avoiders (0 serves), high consumers (6+ serves/day) were: more likely male and socially advantaged, had a healthier dietary pattern, less likely dieting, overweight or obese, and were at lower risk of metabolic complications. After adjustment for age, sex and energy intake, there was an inverse relationship between core grain serves intake and BMI (*p* < 0.001), waist circumference (*p* = 0.001) and a positive relationship with fibre (*p* < 0.001). Model adjustments for diet and lifestyle factors resulted in a smaller difference in waist circumference (*p* = 0.006) and BMI (*p* = 0.006). Core grain serves was significantly associated with higher fibre, but marginally clinically significant for lower adiposity.

## 1. Introduction

In 2014, 600 million people worldwide were classified as obese [1], and the number of overweight Australians has increased dramatically over the last 20 years [2]. In the latest health survey, 63% of Australian adults were overweight or obese and those aged 65–74 years were at the greatest risk, with 75% overweight or obese, compared with 36% of 18–24 year olds [3]. As obesity is largely preventable with adequate diet and physical activity interventions [1,2], we need evidence-based dietary strategies for weight management that are affordable and accessible to populations.

One such strategy is the consumption of grain foods. Grains are nutrient rich, providing carbohydrates (energy), protein, fibre and a wide range of vitamins and minerals including folate, thiamine, riboflavin, niacin, iron, vitamin E, zinc, magnesium, phosphorus and phytonutrients [2]. There is increasing epidemiological evidence that consumption of grain foods is associated with less weight gained over time [2,4,5,6]. Specifically, the higher fibre and whole grain foods reduce the risk of weight gain [7,8,9,10,11], and consequently reduce population burden diseases such as type 2 diabetes, cardiovascular disease, obesity and some cancers [7,8,12,13,14]. Most cross-sectional studies focus on the relationship between whole grain intake and anthropometric measures [11,15,16], rather than all grains, and studies show that the inherent high-fibre content of whole grains may assist with weight loss through satiety and the reduction in postprandial glucose [17,18], and that grain derived fibre rather than fruit or vegetable fibre is protective against weight gain [7,8].

The Australian Dietary Guidelines (ADG) recommend eating a variety of nutritious foods every day including the Five Food core group “grain (cereal) foods”. The ADG specifies grain (cereal) foods, herein referred to as “core grain foods”, should be mostly whole grain and/or high cereal fibre varieties, and includes both refined and wholegrain varieties of; breads, cereals, crispbreads, rice, pasta, noodles, polenta, couscous, oats, quinoa and barley. While the Australian core grain foods definition includes some refined grain foods, such as white rice and white breads, it excludes other refined grain foods such as; cakes, muffins, pastries, biscuits and pizza. These excluded grain foods are described by the ADG as discretionary foods as they lack whole grains, are low in fibre and typically have high added sugar, fat and/or salt [2]. 

It is important to investigate intake of complete food groups as recommended by national guidelines, such as core grain foods (whole grain and some refined grain foods). There is little information worldwide regarding consumption of these core grain foods as a whole food group and there are no nationally representative data in Australia. Many studies focus on either whole grain or refined grain food intakes, however, this does not reflect national guidelines for grain food consumption, nor how individuals consume the whole food group of grains as a mixture of whole and refined. The literature on whole grain intake and weight measures is limited by the varying definitions of “whole grain food” [19,20]. Further, many studies do not adjust for socio-economic status (SES), dieting and under-reporting despite evidence of potential interactions with weight status [8,11,12,21]. There is a need to understand if the consumption of core grain foods is associated with higher fibre intakes, despite its refined grain content, and its influence on weight status.

Thus, the aim of this study was to determine the association between the consumption of core grain food serves and its effect on fibre intakes and anthropometric measures in a nationally representative sample of Australian adults while adjusting for important covariates that can influence fibre intake and weight status.

## 2. Materials and Methods

### 2.1. Study Population

The 2011–2013 Australian Health Survey (AHS) was conducted by the Australian Bureau of Statistics (ABS) and includes a nationally representative sample of 35,000 Australians (>2 years). The AHS incorporated the National Nutrition and Physical Activity Survey (NNPAS) and collected detailed physical activity information using self-reported and pedometer collection methods, along with detailed information on dietary intake and foods consumed. A 24-hour dietary recall questionnaire collected detailed information on all foods and beverages consumed on the day prior to interview, from midnight to midnight, using the Automated Multiple-Pass Method developed by the Agricultural Research Service of the United States Department of Agriculture, and adapted to reflect the Australian food supply. Total energy and dietary fibre intakes were derived from the Food Standards Australia New Zealand customised nutrient composition database [22]. We analysed data from the 9341 adults (>18 years) in the NNPAS. Further survey details are available on the ABS’s website under Australian Health Survey: Users’ Guide, 2011–2013 [23].

### 2.2. Discretionary Foods

Foods were coded as discretionary or non-discretionary in the NNPAS by the ABS and defined based on a combination of descriptions used in the Australian Dietary Guidelines (ADG) [2] and nutrient profiling [23]. The ADG describe discretionary foods as: “foods and drinks not necessary to provide the nutrients the body needs, but that may add variety. They are high in saturated fats, sugars, salt and/or alcohol, and are therefore not a necessary part of the diet.” Grain foods that were defined as discretionary in this survey included: bread rolls topped with cheese, sweet buns, garlic bread, French toast, highly pre-sweetened breakfast cereals, sweet biscuits, some savoury biscuits, cakes and cake-style muffins, slices and brownies, puddings, some scones, sweet and savoury pastries and pies, quiches, savoury dumplings, spring and sausage rolls, samosas, hot dogs, nachos and tacos, waffles and doughnuts. For mixed dishes with a grain component, such as sandwiches, burgers, wraps, sushi, and pizza, those with >5 g saturated fat per 100 g were defined as discretionary.

### 2.3. Grain Definition and Serves Calculation

The ADG core food group “grains (cereals)” includes: breads; breakfast cereals; crispbreads; grains (e.g., wheat, rice, oats, quinoa, barley, buckwheat); and grain products (e.g., pasta, noodles, couscous, bulgur, semolina, polenta, popcorn, flour) [2]. It does not include discretionary grain foods (e.g., cakes, pastries and biscuits). Thus, core grain foods were classified as the two major food groups: “cereals and cereal products”; and the “cereal based products and dishes”. These food groups also included some non-grain foods; such as sago, tapioca, gluten-free flour (non-grain-based) and arrowroot flour. In order to capture core grain food consumption among Australians, discretionary and non-grain foods were excluded from these food groups. This analysis does not include grain foods that are reported as a minor ingredient in mixed foods (e.g., salads with minor grain ingredients, or mixed dishes with meat as the major ingredient but also containing grains).

The ADG serve sizes for core grain foods, in grams were calculated at the 8-digit food code level (the highest level of information given in the AUSNUT 2011–2013 database) [22]. The serve sizes for food categories which correspond to core grain foods were defined using the serve sizes published in the 2013 ADG [2]. These included: 1 slice of bread, 1/2 medium bread roll, or 1/2 medium flat bread (all 40 g); 1 crumpet (60 g), 1 small English muffin or scone (35 g); 1/2 cup cooked porridge (120 g), 2/3 cup of cereal flakes (30 g), 1/4 cup of muesli (30 g); 3 crispbreads (35 g); 1/4 cup of flour (30 g). For uncooked or undrained plain grain products (excluding mixed meals), where a cooked/drained serve size was defined by the dietary guidelines, the serve size of the uncooked and/or undrained product was calculated based on an energy equivalent to 1 serve of the cooked product using the survey’s database: AUSNUT 2011–2013. For foods not clearly assigned a serve size in the dietary guidelines, the same serve size outlined in the modelling of the guidelines was used. For foods where grains were the main ingredient in a mixed dish, the AUSNUT 2011–2013 food recipe file was used to calculate the core grain food content within the food recipe: the amount of that food required to deliver 1 serve of core grain food (i.e., for a sandwich with crumbed chicken, the bread and the bread crumbs were included in the calculation of the amount required to deliver 1 serve of bread). Where a recipe for a mixed dish was not provided, the serve size was estimated based on a similar food within the food group. Mean serves were calculated for each person and respondents were categorised into four categories of core grain consumption to align with the ADG recommendations: >0–<2, ≥2–<4, ≥4–<6, 6+ serves. Core grain food avoiders had zero (0) serves of core grain foods, though they may have consumed discretionary grain foods.

### 2.4. Exposure Variables

Demographic variables included age (19–30, 31–50, 51–70, 71+ years), sex, socio-economic sttus (SES), education and anthropometric measures. SES was defined based on Socio-Economic Indexes for Areas (SEIFA) [23]. SEIFA is a product developed by the Australian Bureau of Statistics that ranks areas in Australia into quintiles according to relative socio-economic advantage or disadvantage. The lowest quintile corresponds to the most disadvantaged, and the highest quintile least disadvantaged. Education was assessed based on the level of the participants’ highest non-school qualification including: postgraduate degree, graduate diploma/certificate, bachelor degree, advanced diploma/certificate, certificate I to IV, certificate not further defined, other education, or secondary school studies only. Anthropometric measures included body mass index (BMI) and waist circumference. Digital scales were used to measure weight (maximum 150 kg), a stadiometer to measure height (maximum 210 cm), and a metal tape measure (which avoided the risk of the tape stretching) to measure waist circumference (maximum 200 cm), without shoes or heavy clothing. BMI was calculated from measured height and weight (kg/m^2^) and adults were categorised as: underweight (<18.5 kg/m^2^), normal weight (≥18.5 kg/m^2^ to <25 kg/m^2^), overweight (≥25 kg/m^2^ to <30 kg/m^2^) or obese (≥30 kg/m^2^) [24]. Participants were also classified according to their waist circumference and the associated risk of metabolic complications: not at risk (<80 cm females, <94 cm males), increased risk (≥80 cm females, ≥94 cm males), or substantially increased risk (≥88 cm females, ≥104 cm males). The cut-offs for waist circumference risk used were as recommended by the World Health Organisation [25]. As part of the survey, participants were asked whether they were currently on a diet and to rate their health. Whether currently on a diet included: currently on a diet to lose weight, currently on a diet for health reasons, currently on a diet to lose weight and for health reasons, not currently on a diet or not applicable. Self-assessed health included: excellent, very good, good, fair and poor. Physical activity categories were based on duration and number of sessions of physical activity reported during the previous week: inactive; insufficiently active or sufficiently active for health. Participants were asked to report the number of serves of vegetables and fruit they usually ate each day, which excluded fruit and vegetable juices. 

### 2.5. Under-Reporters

Basal metabolic rate (BMR) is the amount of energy needed for a minimal set of functions necessary for life over a defined period of time. BMR is given in kilojoules (kJ) per 24 hours and calculated using age, sex and weight (kg) as variables with no adjustment for activity levels. Energy intake to basal metabolic rate ratio (EI:BMR) was used to classify under-reporters: participants with implausibly low intakes. Participants were classified as under-reporters based on the Goldberg [26] cut-off limit of 0.9 for EI:BMR, which is the lower 95% confidence limit for a single day of data for a single individual, allowing for day-to-day variation in energy intakes, and errors in calculation of EI:BMR.

### 2.6. Statistical Analysis

Data were weighted to match the Australian population in such a way that the total weighted sample size was 9341. The statistical package SPSS version 21 was used for all analyses. Due to the large sample size, *p*-values < 0.001 were treated as significant, while values between 0.001 and 0.01 were treated as marginal; thus, very small differences are not declared to be “significant” simply due to the sample size. Descriptive summaries were calculated for all variables of interest. Bivariate analyses investigated the relationship between core grain consumption group and categorical variables using chi-squared tests, and numerical variables using one-way analyses of variance. 

General linear models were used to investigate the effect of core grain food consumption (0, >0 to <2, ≥2 to <4, ≥4 to <6, 6+ serves), on three key response variables: waist circumference (cm), BMI (kg/m^2^) and dietary fibre intake (g). These models included adjustment for important background variables. Model 1 included sex, age group (19–30, 31–50, 51–70, 71+ years) and total energy intake. Model 2 added a range of factors and covariates: SES, education, dieting, self-assessed health, physical activity, percent of energy from discretionary foods and usual fruit and vegetable intake. Model 3 included the indicator variable for under-reporting (0– not an under-reporter; 1– under-reporter).

These models resulted in ANOVA tables showing which variables were significant, parameter estimates, tables of marginal means, post hoc pairwise comparisons using the Bonferroni correction for multiple testing, and profile plots.

## 3. Results

### 3.1. Descriptive

A large majority of the population consumed core grain foods (94.3%), 19.2% had 6 or more serves and 5.7% did not report any serves of core grain foods. Core grain food consumption ranged from 0 to 22.5 serves, with a mean of 4.0 serves (median 3.4 and quartiles of 2.0 and 5.4 serves). In comparison with lower consumers of core grain foods and core grain food avoiders, high core grain food consumers (6 or more core grain food serves) were the most likely to be male, well educated, of higher SES, to have rated their health as excellent, had higher physical activity levels, and the least likely to be on any form of a diet (all with *p* < 0.001) (Table 1). They were slightly younger than all except core grain food avoiders, had a considerably higher fibre and total energy intake than all other respondents, but lower discretionary energy intake than all except consumers of 2 to <4 core grain serves, and were the least likely to under-report their energy intake (all with *p* < 0.001). In comparison with all core grain food consumers, core grain food avoiders were the youngest, least likely to be well educated, most likely to be overweight or obese and to be dieting, had the lowest fibre and total energy intake but highest discretionary energy intake (including discretionary grain foods), and were the most likely to under-report their energy intake (all with *p* < 0.001). Core grain food avoiders had a higher absolute mean energy intake from discretionary grain foods (1104 kJ) than core grain food consumers (829 kJ), and a higher percent of energy intake from discretionary *grain* foods (14.1%) than core grain food consumers (8.8%). They had the lowest self-reported usual consumption of fruit and vegetables, which together with their avoidance of core grain foods resulted in the lowest intake of fibre. They were also the most likely to under-report their energy intake.

### 3.2. Modelling Results

Model 1 for waist circumference, BMI and fibre consumption had an *R*-squared values of 21%, 6.5% and 36% respectively (Table 2). Sex, age group and serves of core grain foods were significant factors in all the models, and total energy intake was significant for BMI and fibre consumption but not for waist circumference. Males had a larger waist circumference, higher BMI and lower fibre intake than females. Increasing age was associated with a larger waist circumference and BMI (with a small decrease in the highest age groups), and a higher fibre intake. Increasing core grain food serves was associated with a smaller waist circumference, lower BMI and higher fibre intake (*p* < 0.001 for trend), though the zero core grain serves (core grain food avoiders) group had a similar waist circumference to the consumers of 6 or more core grain food serves.

Further analyses in Model 2 included adjustment for a range of factors (education, SES, self-rated health, being on a diet for weight loss or health reasons, physical activity level) and covariates (percent energy from discretionary foods, and usual serves of fruit and vegetables). The model for waist circumference had an *R*-squared of 30%. Sex and age group were significant (*p* < 0.001), and core grain food serves had small but significant interaction with age group (*p* < 0.001) and a marginally significant interaction with sex (*p* = 0.006). The general pattern was an increase of waist circumference with age, particularly from the 19–30 years to 31–50 years, and continuing to increase in the 51–70 years; however, the core grain food avoiders (the zero serve group) showed a distinct drop in waist circumference from 31–50 years to 51–70 years. This pattern was observed in both males and females, though with small variation between the sexes. All the additional factors and covariates were significant (*p* < 0.001, usual vegetable serves *p* = 0.006, marginal) except for total energy intake (*p* = 0.84) and serves of fruit (*p* = 0.73). The conclusions from Model 1 about the effects of sex, age group and core grain food serves were confirmed.

The model for BMI had an *R*-squared of 16%. Sex and age group were significant (*p* < 0.001), and core grain food serves had a marginally significant interaction with age group (*p* = 0.006). BMI values were substantially lower for the core grain food avoiders at the higher ages; the drop from 31–50 years to 51–70 years is particularly noticeable and contrasts with the rise for all other core grain food consumption groups. In women, BMI values were the highest among core grain food avoiders up to 50y. All the additional factors and covariates were significant (*p* < 0.001) except for total energy intake (*p* = 0.12), percent of energy from discretionary foods (*p* = 0.28) and serves of fruit (*p* = 0.35). The conclusions from Model 1 about the effects of sex, age group and core grain food serves were confirmed.

The model for fibre consumption had an *R*-squared of 49%. Age group and core grain food serves had significant main effects (*p* < 0.001), showing an overall rise in fibre consumption with age and with increasing core grain food consumption. Sex and core grain food serves had significant interaction effects with age group (*p* < 0.001). The lowest core grain food serves groups (core grain food avoiders and consumers of less than two serves) went against the trend of increase in fibre over age; for males the increase was only slight, while for females there was a slight falling trend. Additional factors and covariates, including total energy intake, were all significant (*p* < 0.001), except for education (*p* = 0.05), self-rated health (*p* = 0.03) and SES (*p* = 0.04). Means and standard errors for main effects were consistent with Model 1 (Table 2). The sex by age group interaction resulted in stronger increases in means across the age groups for males (mean ± SE: 21.6 ± 0.4, 23.1 ± 0.3, 24.1 ± 0.3, 25.9 ± 0.6 g) than for females (23.6 ± 0.4, 23.1 ± 0.3, 24.2 ± 0.3, 24.9 ± 0.6 g). 

In Model 3, under-reporters were included as a factor (Table 2). Under-reporters were more likely to be found in the higher BMI groups (prevalence of 12%, 14%, 21% and 34% among underweight, normal, overweight and obese groups, respectively) and they reported around half the core grain food serves of the non-under-reporters. As a result of including the variable for under-reporting, the *R*-squared for the model for waist circumference increased to 33%, for the model for BMI to 21%, and for the model for fibre it remained at 49%. The overall results from these models were consistent with those from model 2, though the strong dependence between BMI categories and under-reporting (*p* < 0.001 from chi-squared test) reduced the significance of core grain food serves in the model for BMI to *p* = 0.11, though its interaction with sex was marginally significant (*p* = 0.006).

**Figure 1:**
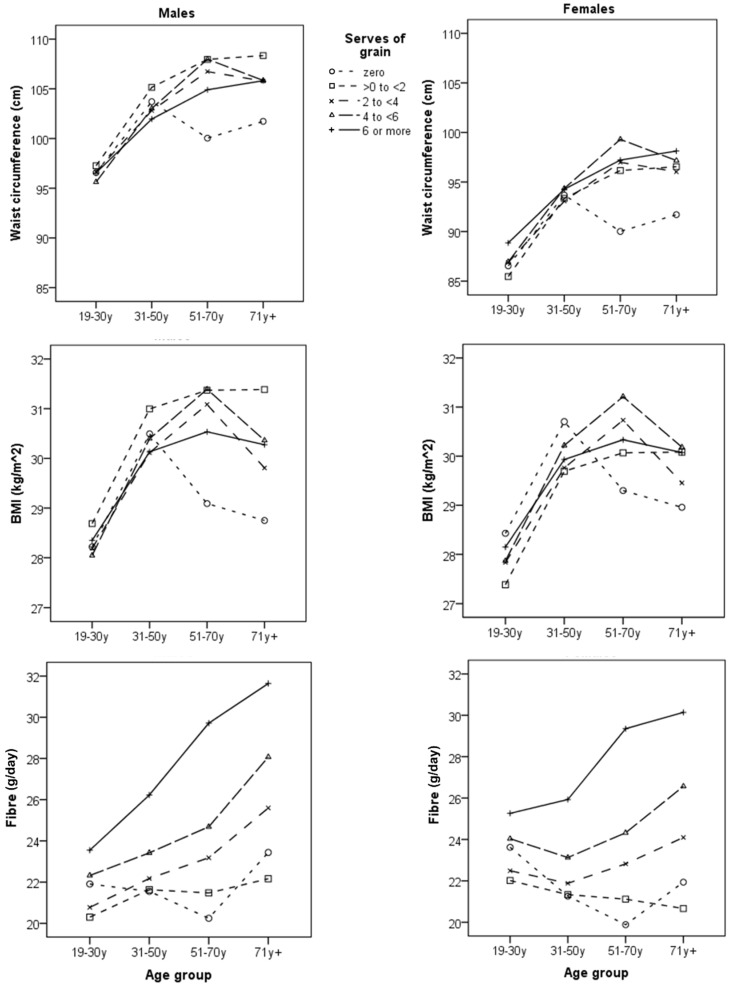
Estimated marginal means from models for waist circumference, BMI and fibre by age groups and sex (Model 3). Covariates in the models evaluated at energy intake (MJ) = 8.8, usual fruit intake serves = 1.62, usual vegetable intake serves = 2.36, percent of energy that is discretionary (%) = 32.7).

The results of Model 3 are shown graphically in Figure 1. Note the difference in waist circumference and BMI for core grain food avoiders at the higher ages (51–70 and 71 years+)—a decline rather than a rise between ages 31–50 years and 51–70 years for core grain food avoiders.

## 4. Discussion

This study aimed to determine if the consumption of core grain food serves as stipulated by the ADG was associated with total dietary fibre intakes and anthropometric measures (waist circumference, BMI). We found that overall, high consumers of core grain foods had a healthier diet and lifestyle pattern compared to core grain food avoiders and were less likely to be overweight or obese. This is despite the fact that the multivariate models showed avoiders 51 years and over had a lower mean waist circumference and BMI than high core grain food consumers. For all multivariate models investigated (for waist circumference, BMI and fibre), groups of core grain food serves, sex, and age were (at least marginally) significant, either as main effects or in interaction (at least marginally significant *p* between 0.001 and 0.01).

### 4.1. Core Grain Foods and Fibre

We found that consumers of high core grain food serves had greater total dietary fibre intakes than low consumers or core grain food avoiders. Core grain food avoiders had the lowest fibre intakes, in all adjusted models. This relationship remained significant irrespective of adjustment for covariates that can influence fibre intake, such as total energy intake, usual fruit and vegetable intake, discretionary energy intakes and other socio-economic and lifestyle factors including educational attainment, physical activity, self-defined health status. Grain (cereal) foods are major sources of dietary fibre, not just whole grain varieties. Evidence from large population studies from the US, Australia and New Zealand show grain foods including rice, pasta, breads, breakfast cereals and other products made of flour, are the largest contributors of total dietary fibre- this ranges from 44% to 47% [3,7,27,28]. The largest food group contributors to fibre intakes are breads and breakfast cereals, in Australia and the US [3,29].

The ADG recommend women aged 19–50 years and men 19–70 years consume a minimum of 6 serves of core grain foods per day, mostly whole grain and/or high cereal fibre varieties. Older women (51 years +) and men (71 years +) have lower recommendations, a minimum of 4 and 4.5 core grain food serves per day, respectively. In this nationally representative sample of Australians, those who consumed greater than 4 serves per day had mean fibre intakes that were substantially higher than those who consumed fewer core grain food serves, despite marginal differences in usual fruit and fibre vegetable intakes. Thus, focused messages on limiting intake of discretionary grain foods and choosing whole grain and higher fibre core grain foods might be necessary for helping people meet fibre targets.

Recent results of the Longitudinal Study of Australian Women’s Health (*n* = 18,226) showed women are falling well short of the recommended daily intake of grain (cereals) foods [30]. Measuring adherence to ADG recommendations for all five food groups, the grain (cereal) group intake was the second lowest. The median intake was 3.3 serves per day and only 7.1% of young women met the recommended serves of cereals of 6 serves per day, but at the older age groups where the recommendations are lower (4 serves), 45% of middle-aged women met the guidelines. Similar to our study, women were less likely to be in the higher groups of core grain consumption and more likely to be core grain food avoiders (54%) [30].

We found that a small proportion of the population avoided core grain foods (i.e., bread, pasta, rice) (5.7%), but were consumers of discretionary grain foods (i.e., pizza, cakes, muffins and biscuits). These people were more likely to be dieting and obese and had poorer diets, high in discretionary foods and low in fruit and vegetables. Even in the model with these covariates included, fibre intake remained compromised. Other studies also show that non-consumers of *whole* grains have significantly lower fibre intakes [12]. Further, avoidance of core grain foods may be related to an increase in popularity of gluten avoidance. Self-reported gluten avoidance in the Australian National Nutrition Survey (2011–2012) was only 2% [3] but wheat and gluten avoidance appears to be becoming popular in Australia [31]. Research suggests that more Australian adults (predominantly female) avoid wheat or gluten than clinically necessary [32]. Golley et al. found that 10.7% of respondents were avoiding wheat (bread, pasta and breakfast cereals were the most avoided foods) while only 1.1% of these respondents were classified as having Coeliac Disease [31]. In a 12-month prospective cohort study of 105 adults diagnosed with Coeliac Disease, Shepherd et al. found nearly all participants had low fibre intakes [33]. Similarly in the UK, a small sample of diagnosed Coeliac sufferers on a gluten-free diet (*n* = 93) had higher sugars and lower intakes of fibre and minerals compared to that of two nationally representative samples [34]. Further research to investigate diet and lifestyle profiles of core grain food avoiders would be beneficial for targeted nutrition promotion, albeit the small number of individuals, because dieting and food group avoidance can result in long-term nutrient inadequacies.

### 4.2. Core Grain Foods and Anthropometric Measures

We found a clear relationship between core grain food serves and anthropometric measures: core grain food avoiders had the lowest measures, and among core grain food consumers, both BMI and waist circumference decreased with increasing serves, even though energy intake also increased. When adjusted for age, sex and energy intake (Model 1), both BMI and waist circumference decreased with increasing core grain food serves. After adjustment for the potential confounders, the difference was clinically small and marginally significant for waist circumference in interaction with age and sex and marginally significant for BMI in interaction with sex. As opposed to the trend found among core grain consumers, avoiders had the lowest anthropometric measures when adjusted for their less healthy diet, lifestyle and under-reporting (which is prominent among the overweight).

Much of the evidence on grain food intake and anthropometric measures specifically investigate *whole* grain consumption; although a relationship with total grains (both refined and whole grain) is also evident. A large cohort from the Netherlands reported that all grains, excluding discretionary grains (in grams per day calculated as the sum of bran, wheat germs, muesli, porridge (oats or whole wheat), brown rice and cooked grains discretionary grain foods) as well as whole grains were associated with a lower odds ratio of being overweight and obese [15]. At baseline, the authors also found lower overall grain consumption among overweight and obese men and women. In our study, core grain food intake, which excluded discretionary grain foods may have been a marker for whole grain intake. A similar multivariate linear regression analysis of NHANES data over 12 years showed that whole grain intake was inversely associated with both waist circumference and BMI among adults [35]. Several reviews investigated the evidence of whole grain consumption on measures of adiposity [4,6,36,37] and concluded that a diet high in whole grains is associated with a lower BMI, waist circumference and risk of being overweight. The prospective cohort studies show a lower gain in weight over time with higher whole grain intakes [8,9,38,39]. Cross-sectional studies show that higher whole grain intakes are associated with significantly lower BMI and waist circumference in both men and women [36]. A recent meta-analysis of randomized trials on whole grain and body weight reduction highlighted that intervention with whole grain only supports the epidemiological evidence within an energy-restricted diet [40]; as previously suggested [36]. Most intervention studies have a shorter duration and perhaps energy restriction is important in the short term for weight loss, while dietary patterns may be more important in the long term for weight maintenance. The mechanism by which grains are associated with weight may be due to its whole grain and/or fibre content or possibly due to reverse causation.

A recent review of epidemiological and intervention studies reported that whole grains over refined grains were beneficial on chronic disease prevention [41]; however, the study identifies a need for a standard definition for what constitutes a “whole grain food”, something that has dominated this field and specific recommendations for observational and intervention studies were recently stipulated [42]. A review on refined grain food intakes and health outcomes showed evidence that up to 50% of grain foods consumed as refined (excluding discretionary grain foods) was not associated with increased disease risk [43]. In our study, we excluded discretionary grain foods from the analysis in order to align with national dietary guidelines. These include foods that are predominantly refined and high in saturated fat and sodium; such as cakes and pizza; and the favourable association between core grain foods and anthropometric measures we observed might be driven by the lack of discretionary foods concurrently with the whole grain content of the diet. Two large cohort studies have shown whole grain to be inversely associated, and refined grain food intake positively associated with weight measures [9,10,12,37]. In the study by McKeown et al., several covariates were adjusted for, and refined grain food intakes were associated with both increased waist circumference and visceral adipose tissue; but those individuals in the highest quintiles of whole grains also had significantly higher intakes of refined grain foods. Other studies found that refined grain foods [39] and refined breakfast cereal intake [38] was not associated with chronic disease risk and weight gain, respectively. Further, in our analysis, the best predictors of both BMI and waist circumference were sex, followed by age, and similarly, the Nurses Health Study showed that BMI increases with age and this is true for both whole grain and refined grain food consumers [9].

Grains are a major source of dietary fibre, and fibre has been shown to assist with weight control [4,7,44,45,46]. The whole grain and/or high fibre grain food content of the diet may independently affect weight status, irrespective of refined grain food intakes, but may be proportionate to *cereal* fibre intakes [11]. Cereal fibre has been shown to protect against weight gain independent of fruit and vegetable fibre [8]. In cross-sectional studies, cereal fibre was inversely associated with BMI and waist circumference [37,47], but not for non-cereal fibre (i.e., vegetable fibre) [47,48]. The postulated mechanisms of dietary fibre include inducing satiety, acting as a mechanical barrier, and its relatively low energy density [44,45,46]. In a recent analysis of trends in intake of whole grain among adults in the US (*n* = 13,276), the main sources of whole grains (breads, ready-to-eat cereals and pastas/cooked cereals/rice) [12] were the same food groups that were main sources of dietary fibre, suggesting that fibre intake may be another mediator of the effect of whole grain on anthropometric measures [37]. 

In our study, we found a clear diet and lifestyle pattern among individuals who were higher consumers of core grain foods. Being cross-sectional, we cannot exclude the possibility of reverse causation and perhaps consumers of core grain foods were generally healthier individuals. High fibre and whole grain consumers generally have healthier lifestyles and are less likely to smoke, be more physically active, and have a better diet quality [4,21,47]. Conversely, diets high in discretionary foods and fast food are associated with weight gain and obesity risk [5,49]. Similar to our results, when studies adjust for diet and lifestyle covariates, the relationship between whole grain intake and weight measures is attenuated [8,37,38,50]. Similarly, we found clear relationships between core grain food intake and lower BMI and waist circumference when adjusted for age, sex and energy intakes (Model 1). When we included multiple covariates in our multivariate model: diet, health and lifestyle factors, and under-reporting, the results were attenuated. In the Netherlands cohort study, when under-reporters were excluded the relationship between whole grain consumption and lower BMI remained [15].

Core grain food avoiders had an unhealthy diet and poorer adiposity measures: they were more likely to be overweight or obese and had the highest discretionary intake despite the lowest total energy intake. In the adjusted models, however, their waist circumference and BMI were similar to those of the core grain food consumers and the older age groups had a lower rather than higher BMI and waist circumference with increasing age. In this survey, older Australians (51–70 years) reported the highest prevalence of being on a diet (19% of females and 15% of males) [3], and this age group also had the highest BMI. In contrast, the younger core grain food avoiders (19–30 and 31–50 years), had a similar BMI and waist circumference to the core grain food consumers. Core grain food avoiders were also the most likely to be under-reporters (44%) compared to high core grain food consumers (4%); but they were also more likely to be overweight- a common relationship observed in the literature [51]. Due to the very small sample of core grain food avoiders, further research is necessary to understand these individuals, particularly at different ages.

This is the first study in Australia to investigate the associations between intake of core grain foods, fibre and anthropometric measures. Strengths of this study include a nationally representative sample, with detailed information on a range of diet and lifestyle factors. Despite including several covariates in our analysis, there is always the possibility of residual confounding for factors not included. Few studies adjust for under-reporting and many exclude participants and perform a sensitivity analysis. However, under-reporting was very high among overweight and obese individuals in this study and is common among the obese [51] which would have resulted in over 3000 adults being excluded from our analysis. Adjusting for under-reporting in multivariate models is recommended [52]. There is also the possibility that many adults were actually on an energy-restricted diet, rather than under-reporting their energy. Core grain foods were widely consumed by nearly all individuals and it is thus less likely to have been specifically underreported.

A limitation of the analysis is the inability to profile the proportion of core grain foods that are whole grain or refined grain. At the time of this analysis, there was no whole database in Australia, but this has recently been published [53]. Methods to estimate whole grains differ across the literature [42]. However, discretionary grain foods were excluded in line with national dietary guidelines. Further, there is the possibility that fortification of refined grain foods with fibre and/or nutrients may play a favourable role in the prevention of obesity. Our study allows us to interpret consumption patterns relative to the ADG, where grams of core grain foods have been converted to serves. Further research is needed to understand the anomalous behaviour of core grain food avoiders including their intake of other food groups from the dietary guidelines, such as dairy, that is sometimes avoided by people avoiding wheat or gluten [31]. It would be useful to profile whole grain and refined grain intakes based on core grain and discretionary grain food, to better understand the possible mechanism between core grain foods and weight status. Further, we used one day of dietary recall to estimate means. The rationale for this approach was that only 65% of adults provided a second day of recall in this survey and because of the large number of covariates included, it was important to retain a larger sample size. 

## 5. Conclusions

In summary, our study showed that Australian adults who had higher intakes of core grain foods had significantly greater fibre intake and lower BMI, and these relationships remained significant after adjustment for several confounders. The dietary and socio-demographic profile of higher core grain food consumers was that they had a healthier lifestyle, were less likely to be dieting, more likely to be male, were more advantaged than consumers with lower core grain food intake and core grain food avoiders, and they were less likely to be overweight, obese or at risk of metabolic complications. Several factors were significant predictors of obesity, including age, sex and number of core grain food serves and further research is needed to better profile core grain food avoiders, especially in older age groups.

## Figures and Tables

**Table 1:** Descriptive characteristics by core grain food serves (mean ± SD, or %).

	Core Grain Food Serves					
	0 (Avoiders) *n* = 535	>0 to <2 *n* = 1809	2 to <4 *n* = 3148	4 to <6 *n* = 2052	6 or More *n* = 1798	*p* *
Sex (% male)	46.4%	37.3%	42.0%	55.6%	68.3%	<0.001
Age (years)	43.6 ± 15.6	45.9 ± 17.2	48.6 ± 18.0	46.0 ± 17.5	43.8 ± 16.9	<0.001
% overweight or obese	66.7%	65.3%	63.7%	61.5%	58.7%	<0.001
% risk of metabolic complications ^1^	37.0%	44.9%	43.1%	38.2%	31.7%	<0.001
Socio Economic Status (highest quintile)	20.6%	20.1%	22.4%	22.8%	23.9%	0.001
Education (Post graduate or Bachelor)	18.5%	26.1%	23.4%	27.4%	29.6%	<0.001
Self-rated health status (excellent)	15.9%	16.0%	15.6%	16.8%	21.7%	<0.001
Physical activity level (high)	16.5%	13.1%	13.2%	14.6%	20.5%	<0.001
Dieting (for any reason)	22.0%	15.0%	14.6%	13.5%	9.1%	<0.001
Total energy intake (MJ/day)	7.11 ± 4.14	7.25 ± 3.32	7.81 ± 3.05	9.25 ± 3.35	11.41 ± 3.63	<0.001
Discretionary energy intake (MJ)	3.58 ± 3.12	3.04 ± 2.76	2.85 ± 2.34	3.12 ± 2.68	2.93 ± 2.53	<0.001
% of energy discretionary	46.2 ± 28.1	37.5 ± 23.3	33.5 ± 19.7	30.5 ± 18.4	24.0 ± 16.0	<0.001
Fibre	15.0 ± 10.5	17.5 ± 9.5	20.4 ± 9.9	24.9 ± 10.9	32.9 ± 13.8	<0.001
Core Grain Food serves	0	1.35 ± 0.46	3.01 ± 0.56	4.92 ± 0.57	8.35 ± 2.41	<0.001
Usual fruit serves	1.4 ± 1.2	1.6 ± 1.1	1.6 ± 1.1	1.6 ± 1.1	1.7 ± 1.2	<0.001
Usual vegetable serves	2.2 ± 1.4	2.3 ± 1.4	2.4 ± 1.3	2.4 ± 1.3	2.4 ± 1.4	0.002
Under-reporters	44.3%	35.0%	25.7%	12.8%	4.4%	<0.001

* *p*-value from one-way ANOVA for numerical variables, chi-squared test of independence for categorical variables; *p*-values < 0.001 significant; between 0.001 and 0.01 marginally significant; ^1^ Based on the World Health Organization cut-offs for waist circumference [25].

**Table 2:** Estimated marginal means (main effects only) for waist circumference, BMI and fibre intake in terms of core grain food serves under three models (mean ± SE).

	Core Grain Food Serves					*p*-Value *	*p*-Value for Trend **
	0 (Avoiders) *n* = 535	>0 to <2 *n* = 1809	2 to <4 *n* = 3148	4 to <6 *n* = 2052	6 or More *n* = 1798		
**Waist circumference (cm)**							
–Model 1	92.1 ± 0.9	94.4 ± 0.4	93.1 ± 0.3	92.9 ± 0.4	91.8 ± 0.4	0.001	<0.001
–Model 2	94.9 ± 0.9	97.6 ± 0.5	96.3 ± 0.4	96.6 ± 0.5	96.3 ± 0.5	0.022	0.30
–Model 3	95.5 ± 0.9	98.8 ± 0.5	98.1 ± 0.4	98.8 ± 0.5	98.5 ± 0.5	0.006	0.53
**BMI (kg/m^2^)**							
–Model 1	27.4 ± 0.4	27.8 ± 0.2	27.2 ± 0.1	27.2 ± 0.1	26.7 ± 0.2	<0.001	<0.001
–Model 2	28.8 ± 0.4	29.3 ± 0.2	28.7 ± 0.2	28.8 ± 0.2	28.6 ± 0.2	0.016	0.12
–Model 3	29.2 ± 0.4	30.0 ± 0.2	29.6 ± 0.2	30.0 ± 0.2	29.7 ± 0.2	0.11	0.49
**Fibre (g)**							
–Model 1	17.2 ± 0.6	19.5 ± 0.3	21.8 ± 0.2	24.5 ± 0.2	29.8 ± 0.3	<0.001	<0.001
–Model 2	21.4 ± 0.6	21.6 ± 0.3	23.1 ± 0.3	24.9 ± 0.3	28.1 ± 0.3	<0.001	<0.001
–Model 3	21.7 ± 0.6	21.3 ± 0.4	22.9 ± 0.3	24.6 ± 0.3	27.7 ± 0.4	<0.001	<0.001

Model 1—with adjustment for age, sex, energy intake; Model 2—Model 1 with adjustment for education, self-assessed health, on a diet, SES, physical activity, percent of energy from discretionary foods, usual serves of fruit and vegetables; Model 3—Model 2 with adjustment for under-reporting; * *p*-value obtained from general linear model; ** *p*-value obtained from general linear model with serves of core grain food used categorically.
